# The Hospital. Nursing Section

**Published:** 1904-10-29

**Authors:** 


### The Hospital.

### Nursing Section.

Contributions for this Section of "THE HOSPITAL" should be addressed to the EDITOR, "THE HOSPITAL," NURSING SECTION, 28 & 29 Southampton Street, Strand, London, W.C.

No. 944.—VOL. XXXVII. *SATURDAY, OCTOBER* 29, 1904.

### Notes on News from the Nursing World.

### THE THANKS OF MISS NIGHTINGALE.

MISS FLORENCE NIGHTINGALE desires to express her thanks for the numerous congratulations which she has received in connection with the jubilee of her departure for the Crimea and which she is unable to acknowledge separately.

### QUEEN ALEXANDRA'S MILITARY NURSING SERVICE.

WE are officially informed that Miss G. A. Magill has resigned her appointment as sister in Queen Alexandra's Imperial Military Nursing Service, and that Miss Nellie Blew and Miss Louisa M. Dann have been appointed staff nurses.

### RUSSIAN NURSES AT THE FRONT.

WHILE complaints are made that the number of Russian nurses at the front is utterly inadequate, it appears that some of those who are there have to put up with indignities, and that certain Russian officers treat Sisters of Charity as they are wont to treat women of the class that follows in the rear of the army. Several nurses are also reported to have died of exhaustion, one of them, a Sister of Charity, while she was assisting at an operation. On the other hand, although it is stated that most of the nurses have behaved with the greatest gallantry and self-sacrifice, a certain number are said to have been ordered back to Russia for unsatisfactory conduct.

### REGISTRATION IN PRACTICE.

IT does not, of course, follow that because an Act of Parliament presses hardly in individual cases it should be condemned. But it seems increasingly clear that registration in practice on the other side of the Atlantic is by no means without serious drawbacks. An American nurse, with a certificate from one of the best known schools, who has held an executive position in one of the hospitals, applied to the New York State Board for registration. The reply of the Board was that "your school does not meet the requirements for registration because it does not afford either practical or theoretical instruction in cooking or materia medica." The nurse was, however, informed that "if the school can advance its requirements so as to afford this instruction it may be possible that it can be registered." Thus the task is imposed upon the nurse of getting her training school to alter its curriculum in order that she may be registered! On the other hand, a nurse whose application to be placed on the register was successful, has frankly stated that, though a graduate of one of the largest training schools in America, "she did not have one day of operating-room work in her whole training!"

### THE STATUS OF THE ENGLISH NURSE.

AFTER the ceremony of the opening of the handsome and admirably appointed New Queen Victoria Memorial Nursing Home at Rochdale had been performed by Lady Derby, an interesting speech was delivered by Lord Derby, who, having dwelt on the nobility of the calling of a sick nurse, mentioned that a compliment had recently been paid to the system of nursing in this country by a French doctor who had visited our shores. He laid stress, Lord Derby added, on the high status of the English nurse, observing that, "whereas English nurses were ladies, in the French hospitals they were servants." It is always pleasant to hear of the superiority of English nurses, but we cannot help doubting whether the interpretation put upon the French doctor's words is exactly what was intended. Seeing that all who serve the sick are servants, we hope that the French doctor merely meant to draw the distinction between those who follow the nursing profession with intelligence and fidelity and those who pursue it simply for the sake of monetary gain.

### THE NEW INSTITUTE IN MADRAS.

THE Committee of the Lady Ampthill Nurses' Institute in Madras found it impossible to secure, as intended, eight nurses who were either trained in India, or, being there, had enjoyed an English training. They advertised in all the leading Indian papers, offering nurses with three years' training 75 rupees a month, and those trained in India 50 rupees, with 8 annas a day extra when out on a case, also board and lodging, and an allowance of 30 rupees for uniform after a nurse had been a year in the institute. As, however, only four nurses in India accepted service on the terms proposed, it became necessary to see what could be done at home, and here no difficulty was experienced in obtaining the remainder. The institute commenced work in July with the matron, Miss Van Coppenolle, and four nurses, who have been almost fully employed since their engagement. We hope that the institute will have a longer and more satisfactory existence than that of its predecessor, the Lady Wenlock Nursing Institute.

### THE POOR-LAW INFIRMARY NURSE AND SURGICAL CASES.

WE notice that Mr. Baldwyn Fleming, Local Government Board Inspector for the counties of Dorset and Hampshire and part of Wilts and Surrey, in his report on Poor-law administration in 1903-4, touches on the training of workhouse nurses. One difficulty, he says, arises from the fact that in most workhouses there is but little surgical work. "In the large town Poor-law infirmaries," Mr. Fleming continues, "there are probably sufficient surgical cases to afford the necessary experience; but in the small places this is not so, and it may be desirable that nurses should be permitted to attend a course of training in surgical work elsewhere. A nurse who has not seen operative surgery cannot claim to be qualified as a surgical nurse. On the other hand, workhouse practice affords a large experience in cases which are not usually met with in general hospitals." This accords with the view which we expressed in commenting last week on the reminiscences of Major Ballantine. We are glad to see that Mr. Fleming resolutely sets his face against the appointment of probationers in small institutions where the circumstances give no promise of efficient training.

### PRINCESS CHRISTIAN AND JOHANNESBURG NURSES.

During the stay of Princess Christian and her daughter, Princess Victoria, in Johannesburg her Royal Highness privately received a number of members of the Royal British Nurses' Association in the Transvaal. Miss Paterson, on their behalf, handed to the Princess a purse which had been collected for her by them in aid of the Helena Benevolent Fund. Subsequently the Princess laid the foundation-stone of the "Bob Stroyan" ward at the Johannesburg Hospital. Before the order of service was begun the matron, the Rev. Mother Adele, and the nursing superintendent, Mrs. Magill, presented bouquets to the Princess, who evinced the keenest interest in everything she saw in the hospital, and expressed great admiration at the artistic decorations which were the work of the sisters and nursing staff. On her departure from Johannesburg her Royal Highness ordered a photograph of herself to be sent to Mrs. Magill.

### NOTICES IN DUTCH HOSPITALS.

Dutch people are very fond of notices and inscriptions, and in the hospitals printed admonitions for the nurses may of[illegible]en be seen. For instance, there is one to the effe[illegible] that "nurses are forbidden to speak in the oper[illegible]ing theatre." In the corridors may be found susp[illegible]ded cards warning probationers "not to rush about in the passages," and to "walk quietly." Such reminders against undue or unnecessary haste and bustle may be excellent in tending to keep nurses gentle. Still they seem hardly necessary in a land where the women are noted for their serenity and common sense. Besides their sound, practical, domestic instincts, the women, in that land of dykes and inundations have been accustomed for many generations past, to face sudden disasters, and to be ready for any emergency, calmly and patiently. They have, therefore, many of the natural qualifications necessary for a good nurse.

### THE NURSE IN THE HOME.

Speaking at the second annual meeting of the Kilburn and West Hampstead District Nursing Association last week, Miss Amy Hughes said that it was a pleasure to find that the work went further than mere technical nursing. The nurses were trained to bring their nursing to bear upon the homes and to teach their patients and those who lived with them how to avoid sickness as well as how to deal with it. She explained how Queen Victoria used the gift of the women of England to found an institution for the training of district nurses. An ordinary hospital nurse was, she thought, rather like a fish out of water in the homes of the poor. Special training was necessary in order to enable her to make the best of poor appliances. In the country the great cry was for maternity nurses, and the country or village nurse received rather a different training from the Queen's nurse.

### A NURSING ASSOCIATION AND AN INDIGNATION MEETING.

The committee of the Barrow District Nursing Association have been accused of getting rid of a nurse because she did not belong to the Church of England, and there has been an indignation meeting at Vickerstown, with severe speeches from the Dissenting minister. It certainly is not, and should not be, the custom of district associations to insist upon nurses being members of any particular church or denomination. As the vice-president of the Barrow organisation explicitly declares that the reason for the nurse leaving had "nothing whatever to do with her religious principles," and that the committee unanimously voted that "her transfer to another home would be beneficial to all concerned," there seems no occasion for religious controversy. In any case, it should be remembered that a nursing association has a clear right to choose its own servants.

### BRAVERY OF AN ETON NURSE.

At the meeting of the Eton Guardians last week a very plucky action on the part of one of the nurses in the workhouse infirmary was reported. An aged inmate having escaped out of the window, the nurse followed him on to the roof, and held him there until assistance arrived. The Guardians warmly commended her conduct, and it was decided to present her with a guinea, subject to the approval of the Local Government Board.

### RESIGNATION OF A MATRON.

We understand that Miss A. A. Gwyn is resigning the post of matron and lady superintendent of nurses at the Royal Hants County Hospital, on account of serious illness in her family. Miss Gwyn was formerly sister at the Royal Hants Hospital, where she received her training, and she has since held the posts of sister at Netley, matron of the General Hospital, Birmingham, matron of the General Infirmary, Worcester, matron of the Bath Hospital, Harrogate, and lady superintendent of the County Hospital, York. The last post she held for more than six years, and she relinquished it in 1900 in order to take up her duties at Winchester. During the time Miss Gwyn has been at the head of the Royal Hants County Hospital Training School she has filled the post with conspicuous ability, devotion, and tact; and we are sure that her retirement will be deeply regretted by all the members of the nursing staff with whom she has always had the happiest relations.

### A RALLY AT WENDOVER.

There has recently been a special meeting in connection with the Wendover Nursing Association for the object of considering its financial position. The organisation has been in existence for nearly three years, and the nurse has gained the confidence of all classes. At the meeting a medical man said that "it would be a thousand pities if her services should be lost through want of funds," and he strongly supported the view that in cases of accidents the people should send for her before sending for the doctor. His speech and others so much impressed the meeting, that several fresh subscriptions were promised at once. Eventually a prominent member of the Association declared that he was now certain that the balance on the wrong side would be made up, and that the unpleasant alternative of doing without a nurse would not have to be further considered. That the poor require one may be gathered by the fact that the number of visits paid in the year exceeds a thousand.

### A COLLAPSE AT RAUNDS.

After an existence of six years, the Raunds Nursing Association has been dissolved because the people in the town will not subscribe £100 a year in order to carry it on. Happily, there are few parishes, either in England or in Northamptonshire, of the size of Raunds, which are so wanting in public spirit. As the result of a special canvass, only £55 was promised, many persons declaring that they could not afford to give, and others saying, more frankly, that they did not feel disposed to do so. A medical man in the town was credited at the winding-up meeting with having asserted that he "did not see the use of a nurse." If the parish be so healthy that it can afford to dispense with a nurse for the sick poor, the services of a doctor can hardly be urgently required, except by the people who can afford to pay for the attendance of a pleasant visitor, whether they are or not in need of medical advice.

### CAMBRIDGE GUARDIANS AND DISTRICT NURSING.

At a meeting of the Cambridge Board of Guardians, a letter was read from the District Nursing Association, asking the guardians to again make their grant of £100 towards the funds of the Association. It was mentioned that a total of 6,497 visits had been paid to Poor-law cases. An attempt to delay the matter by referring it to the Finance Committee was not successful, the majority of the guardians being of opinion that it was the truest economy to support the Association. The clerk was accordingly instructed to write to the Local Government Board for sanction to contribute the same sum as before.

### NURSES' HOME AT VICTORIA PARK.

The first section of the new home for the nurses at the City of London Chest Hospital is almost finished, and will, it is hoped, be ready for occupation by Christmas. It consists of 29 bedrooms and a linen-room, on three storeys, with two bathrooms and lavatories on each floor. The floors will be covered with plain linoleum, and the walls of the bedrooms are to be coloured with a pale green wash. Those on the top floor, which will be allocated to the night nurses, have deep window seats which add greatly to their appearance. In order to avoid the risk of ink-stains in the bedrooms there will be small writing-tables and chairs on the landings, which are light and well-warmed by means of hot-water pipes. It is intended to furnish with as many drawers as possible, and no boxes will be allowed in the rooms. The building is completed by an outside iron staircase for use in case of fire; and, when funds permit, the remaining portion of the scheme will at once be proceeded with, consisting of 10 more bedrooms, sisters' rooms, and nurses' sitting-room, with a subway to connect the entire block with the dining-room and the hospital building. The original estimate of £7,000 will probably be exceeded, the cost, including furnishing and equipment, being nearer £9,000.

### THE QUEEN'S NURSES AT LYNN.

The Mayor and Mayoress of Lynn gave an entertainment in the assembly-rooms of the Town Hall the other evening, for the purpose of assisting the funds of the District Nursing Association which is affiliated to Queen Victoria's Jubilee Institute. The company, as they entered, deposited their money gifts in a silver tray, and the amount realised by the offerings of present or absent donors was nearly £30. The deficit on the accounts for last year was upwards of £50, and it is hoped that the success of the special effort will act as a stimulant to the collectors of the Association, and be the means of augmenting the subscription list. That the two nurses are badly needed in Lynn may be judged by the fact that during the past three months the number of cases had increased by 50 as compared with the corresponding time in 1903, while the number of visits paid during that period was nearly 2,300.

### THE UNIFORM QUESTION AT WORKHOUSE INFIRMARIES.

While the Knaresborough Guardians have unanimously agreed to provide the nurses in their employ with uniform at a cost of £2 10s. yearly, a difficulty has arisen at Bath on the same subject. The Hospital Committee recommended the Guardians to sanction the provision of a distinctive uniform for probationers to be worn out of doors, at a cost of £1 10s. each. This recommendation, however, is not approved by the medical officer and the superintendent nurse, both of whom are opposed to probationers wearing uniform out of doors. The former told the Bath Guardians that he does not consider it fair to trained nurses that probationers should wear outdoor uniform, and eventually this view was endorsed by the Guardians.

### THE ROYAL WATERLOO HOSPITAL.

Although out-patients are being received in the new building of the Royal Waterloo Hospital, and it is hoped that part of the in-patient accommodation will be ready for occupation early in the new year, the prospect of a permanent home for the nursing staff continues to be remote, owing to want of funds. The premises in Stamford Street are still being used as a temporary home for the nurses.

### OUR CHRISTMAS DISTRIBUTION.

We have to acknowledge with thanks the receipt of contributions to our Christmas distribution of articles of clothing from Miss Mary Besant, 4 York Street Chambers, London, W., and Policy Holder 8659, as well as a second parcel from M. R., Ryde. All parcels should be addressed to the Editor, 28 and 29 Southampton Street, Strand, London, W.C., with "Clothing Distribution" written upon the outside.

### The Nursing Outlook.

"From magnanimity, all fear above;
From nobler recompense, above applause,
Which owes to man's short outlook all its charm."

### NURSES FOR THE MIDDLE CLASSES.

We published on the 15th of this month an interesting interview with Miss Thorne on the subject of visiting nurses for the middle classes, a notice having been circulated that a benefactor was going to start a scheme for supplying nurses to the fairly well-to-do. It is an undoubted fact that the middle class in England is increasing constantly in numbers, and that its needs do not receive proper consideration. If the clerk or the poor clergyman seek the shelter of the hospital, we have columns about "hospital abuse"; if they ask to be allowed to pay what they can afford, they are told they must either pay the full fees of a nursing home or accept the charity of the common wards. And the services of the district nurse is denied them.

Under these circumstances a certain number of women have started as visiting nurses on their own account, and have done good work in certain restricted areas; but inasmuch as the movement was not general, and was not widely known, their work has been limited. It is now proposed to have "homes" from which these nurses should work, and though they are to charge moderate fees, their incomes are to be eked out by the hands of the benevolent, and they are to work under committees, and be subjected to inspection. In fact, they are to be run on the lines of the socialist motto: "To each according to his needs; from each according to his powers." And, indeed, this is the only way of equalising things in this century of classes and masses. We have got to realise that the world is no more divided into rich and poor than it is into good and bad, but that the majority are "medium" in all things; are neither rich nor poor; are neither good nor bad. In continental towns there is generally some public hospital accommodation for the patient who wishes to pay according to his means; indeed, in the huge hospital at Hamburg there are about a dozen pavilions which receive all, from the very rich to the absolutely poor, and all have the same medical treatment, though the pavilion for the aristocrats is more daintily furnished and has a larger percentage of service than the other divisions. Something of this sort is badly needed in London; some half-way house between charity and the expensive nursing home in the neighbourhood of Cavendish Square; and until we have it we could gladly welcome an extension of the movement for supplying visiting nurses.

There are so many cases in which the constant presence of a stranger would only mean restraint and worry, and where the illness demands but a morning and evening visit, the question of fees may be an important one; still, it is not only the smaller charge that makes the visiting nurse popular, but the fact that she is not such a nuisance to the patient, not such a burden on the household. In all these huge houses filled with flats, in all these rows of mean suburban houses, there may be no extreme poverty, but there is no room for a nurse. The young man in chambers, the young woman in her tiny flat, are subject to attacks of influenza and rheumatism like the rest of us; they are probably living alone, they earn their living by some small weekly wage—a wage very likely not so high as that of a mechanic; but they shrink from seeking the hospital, they have no brother nor sister at hand to help them, and they cannot house a resident nurse, nor can they afford her fee, nor supply her many requirements. It were, indeed worth while then to organise for all London, and for all big towns, nurses of skill and character who could be trusted to visit such cases daily, to take a moderate fee, and to advise, if necessary, removal to a hospital, or the securing of the services of a permanent nurse.

The work would commend itself very kindly to our elderly and sober nurses, who have lost the feverish desire for the excitement of the operating theatre, and the longing to be engaged constantly in a hand-to-hand fight with death. The strain of nursing work was so great a few years ago that a theory got abroad that a nurse over fifty was quite out of date. Now we have altered all that; we have come to recognise that the young nurse has her limitations and that the world is big enough to offer work to all, and that the nurse of sixty even has her place in the universe. And for the nursing of the middle class, neither the very young nor the very old should be chosen; the responsibility will be great and the work will be trying. It needs a steadfast worker and a sober woman to visit successfully in the flats and chambers and small houses in London. There are some nurses who go to district work who find before long that their sympathies become dulled by constant contact with sordid poverty, that the dirt and the vermin fill them with such disgust that they lose that sweet serenity of soul which is the core of all good work. However much they may pity their patients they cannot do the work cheerfully and well, and their powers are blunted. Such women are eminently fitted for nursing the middle classes; there the refined economies, the hidden needs, call forth their sympathy without jarring on their susceptibilities; it seems to them much more pitiful that the girl typist cannot afford meat every day than that the dock labourer should go breakfastless to his task. If it be true "to each his sufferings," it is also true "to each his sympathies," and we feel confident that once this organisation for supplying visiting nurses is developed, several of our readers will there find their true sphere of work, and render excellent service.

### Lectures upon the Nursing of Infectious Diseases.

By F. J. Woollacott, M.A., M.D., B.S.Oxon, D.P.H., Senior Assistant Medical Officer, Park Hospital, Metropolitan Asylums Board, Hither Green.

LECTURE VIII.—DIPHTHERIA (*continued*).

In the treatment of a case of diphtheria the first essential is the administration of antitoxin. This part of the subject will be considered subsequently.

What was said in the lecture on the complications of scarlet fever concerning the treatment of the throat and adjacent parts applies in almost every particular to diphtheria, although local treatment is less necessary on account of the valuable help rendered by the antitoxin. Syringing or irrigation of the fauces, nose, and ears may be employed to wash away discharges and restore the parts to a healthy condition. The greatest gentleness should be used, otherwise injury is almost certain to result. If a piece of membrane is seen to be hanging loose so as to cause the patient annoyance it may be carefully detached with forceps, but for the most part syringing will do all that is necessary. In some cases a complete cast of the nasal cavities will be washed out, its removal always being followed by great relief. It frequently happens that as the membrane separates a sharp attack of bleeding occurs, usually from the nose. This is readily checked by the application of ice to the forehead and bridge of the nose, or by the use of an iced lotion for syringing. Fomentations or poultices to the neck are often beneficial and help to subdue the inflammation. As a rule, with this treatment, and the injection of antitoxin the local conditions rapidly improve. If the temperature be much raised, or if the patient be restless, tepid sponging will be found useful. The food in the acute stage should be light and easy of digestion. If the case be at all severe liquids alone should be given, in small quantities and at intervals of about two hours. Milk will be found to be most generally useful, diluted, if necessary, and suitably flavoured. Owing to the pain and discomfort of swallowing it is sometimes difficult to get the patient to take enough nourishment, but much may be done by tact and perseverance on the part of the nurse. Nasal feeding is often impossible, as the nose may be blocked by membrane, and the passage of the tube may give rise to troublesome bleeding. Care must be taken to avoid anything likely to cause vomiting, and with this object in view it is better to keep the bowels open with enemata of glycerine or warm water, rather than with aperients. As the throat clears, the diet should be increased and made as nourishing as possible in order to restore and maintain the patient's strength. The digestion, however, is frequently impaired, and the appetite poor, so that the increase should be made gradually and cautiously. Large meals should be avoided; they overload the stomach, and in consequence may set up vomiting or, perhaps, embarrass the action of the heart.

If vomiting occur the food must be given in very small quantities only, and peptonised milk and similar preparations may be tried. Unfortunately, in many cases the stomach rejects nourishment in any form, and rectal feeding is our only resource. With care, it is often possible to keep the patient alive by this means until food can be taken in the natural way. Success depends largely on attention to detail. An enema of warm water should be administered first in order to empty the lower bowel, and each day this should be repeated, the object being to wash out the rectum, and remove the unabsorbed débris. Unless this be done, irritation is soon set up and diarrhœa results. The feed should, as a general rule consist of peptonised milk, 3 or 4 oz. of which may be given every four hours in the case of young children.

It is best to use a soft catheter and a funnel. The former is to be oiled, and can then be easily passed into the rectum for a few inches. The fluid should run in slowly, and care must be taken to prevent the entrance of air. The greatest gentleness is essential. Should the enema be badly retained, or show a tendency to return at once, a pad may be pressed against the anus for a few minutes and the buttocks raised on a firm pillow or the foot of the bed tilted up. If the sphincter of the anus be weakened and relaxed, as sometimes happens in the course of paralysis, it may be almost impossible to prevent the enema from returning, whatever devices be adopted. Such cases are, however, rare, and by attention to the directions just given, life may be sustained for many weeks. The patient will of course rapidly waste, even under the most favourable circumstances, as the amount of nourishment is miserably inadequate.

As we have seen, the chief danger in diphtheria results from the weakness of the heart, and we must consequently do all in our power to strengthen it and to make its work as light as possible. The patient, during the acute stage and for some time after, must remain quiet and of course never be allowed to get out of bed. As far as possible he should be kept lying down. In the case of children, however, this rule can rarely be rigidly enforced, for, unless allowed to sit up occasionally, they become excited and fretful, and harm may ensue. The best plan is to prop them up in bed with pillows if they insist on sitting up, and in most cases they will soon get tired and ready to lie down again.

When symptoms of heart failure or cardiac paralysis show themselves in the early stage of the disease, the outlook becomes very serious, and treatment is frequently unavailing. As the patient is usually restless, is is well to raise and pad the sides of the cot or bed to prevent him from bruising himself or falling out. He should be kept warm with hot bottles, and the clothing should be close fitting, for otherwise it will be thrown off. The pillows should be removed in order to keep the head low, and with the same object the foot of the bed may be tilted up. If there be pain over the heart or abdomen, fomentations, frequently changed, may afford relief. Rectal feeding may be tried. But little help is afforded by drugs, and stimulants are rarely of much use.

When the attack of heart failure occurs later in the disease the prospect is much more encouraging, and the treatment just described will often be attended with success.

The progress towards recovery from diphtheria is usually slow, and every advance must be made cautiously. The times for sitting up in bed, getting up, and beginning to stand and walk, will all need careful consideration, and are chiefly determined by the condition of the heart. Convalescence may be complicated by paralysis, as will be explained in a future lecture. As a rule in the milder cases, and in the absence of complications, the patient is kept in bed for two or three weeks, and confined to the room for a week or two longer. In cases of a more severe type, convalescence may not be established for some months.

The ultimate recovery is nearly always complete in every way, and no permanent ill-effects remain, though anæmia and debility may long continue to cause anxiety. The heart regains strength, and the kidneys sooner or later are restored to a healthy condition. Ear discharge, however, may occasionally become chronic, and even lead to permanent deafness.

The length of time during which a case may continue to give off infection varies considerably, from a few days to several months.

It should be rembered that infection chiefly resides in the discharges from the nose and throat. Pieces of rag or lint should be used to wipe these away, and then immediately burnt. If by accident the patient coughs into the nurse's face, it should be thoroughly washed with warm water or boracic lotion. This precaution is particularly necessary while nursing a case of tracheotomy. Sore throat, however slight, should always receive medical attention at once, as the onset of the disease is often insidious and early treatment of paramount importance.

*Other Forms of Diphtheria.*—In addition to the common form of diphtheria, other and rarer forms are sufficiently important for brief description. Croup will be considered subsequently. Occasionally the disease remains localised in the nose and does not spread to the fauces. The nasal cavities are lined with membrane, and a discharge, more or less offensive, results.

The mucous membranes in other parts of the body may be involved, usually in consequence of accidental infection from the throat. This is, perhaps, most common in the vulva. It appears considerably swollen, and on its inner surface a dirty grey membrane forms. In rare instances the process may attack a wound or sore of the skin, and give rise to similar symptoms.

As in any other form of diphtheria antitoxin should always be given, and this, with cleanliness and the application of fomentations, or some other simple dressing, will rapidly effect a cure.

More serious mischief may follow diphtheria of the conjunctiva which, in the absence of prompt treatment, may cause ulceration of the cornea or even total destruction of the eyeball. The symptoms are swelling of the eyelids, with formation of thin membrane on their inner surface, and more or less copious discharge, together with general inflammation. In addition to antitoxin, treatment consists in washing away the discharge by frequent bathing or syringing, and the application of fomentations.

It must be remembered that all these local forms of diphtheria are infectious, and strict precautions should always be taken.

### Presentations.

Chiddingfold District Nursing Association.—Miss Margaret H. Beeks, who is now matron of the Eden Cottage Hospital, Hatfield Broad Oak, Essex, and who was district nurse at Chiddingfold, Surrey, from March 1901 to May 1904, has been presented by the inhabitants of Chiddingfold with a testimonial and a water-colour painting of the village and church, in happy remembrance of three years' work in the parish.

### TRAVEL NOTES AND QUERIES.

By our Travel Correspondent.

A Winter in the South (Lonsdale).—I shall be pleased to help you when you give me something to go upon. "The South of Europe" is so vague and you give me no idea of what you can pay. Write quite fully saying: (1) What you wish to pay each per week. (2) Do you prefer the seaside or inland; either say the Riviera or Pau? (3) Do your fancies turn most to Italy or to France? (4) What are your tastes? So much depends on that. If you are greatly interested in art or in architecture, ecclesiastical and domestic, you might prefer such places as Rome or Florence, but if you prefer the country on the whole, then I should say either the French or Italian Riviera or Pau, or, perhaps, St. Jean-de-Luz, just on the Spanish frontier. When I know exactly what you can spend each week without feeling extravagant I can give you all particulars.

### The "Medical Register" Swindle.

Our attention has been directed by the secretary of a well known organisation for nurses to a matter of considerable interest It seems that a man has lately appeared in a Kentish suburb of London, where he has been interviewing nurses telling them that "unless they have their names put down in the 'Medical Register' they will be supposed to have no certificate, and will get thrown out of work, as all nurses must now hold certificates." A nurse, who is probably only one of several more who have attached credence to this strange statement, paid 3s. and received the following receipt:—"No. J 1,175, September 17th, 1904. Received from Nurse ——, the sum of —— pounds, three shillings, and —— pence for insertion in 'Medical Register.' For J. Howard and Co., 78 Fleet Street, E.C. W. Morgan." The man told the nurse that she would have to pay another fee in 18 months, that the registration was something "to do with the Government," and that she would hear from him in a fortnight. More than that time has already elapsed. Amongst other assertions he told the nurse that he had seen her name in the books at a certain hospital where she had been trained, and this, of course, impressed her. She had probably let drop this piece of information earlier in the conversation. After being handed the receipt the nurse was told that she "might now put the number which was at the top of the receipt under her name on the brass plate on the door."

Upon receipt of this information one of our representatives called at the address given to make inquiries, but at 78 Fleet Street nothing whatever is known of either J. Howard and Co., W. Morgan, or the "Medical Register." No letters have been received addressed to either of the persons named, or to the "Medical Register"; and it is therefore quite obvious that a false address has been given. For the rest, though it is astonishing that any nurses should be so credulous as to part with their money on the faith of such a cock-and-bull story, we think it advisable to warn our readers against Messrs. J. Howard and Co., or W. Morgan, and if any have been induced to part with money in order to be "registered," we shall be glad to hear from them.

### The Nurses' Home at the Cancer Hospital, Brompton.

The new home for the nursing staff of the Cancer Hospital, Fulham Road, is practically finished, and will be opened on November 14th by Lady Ludlow. The building is of red brick, with four gables. The main entrance occupies the left-hand corner, and from a wide entrance hall with a rounded archway, two corridors radiate to right and left, the rooms being on either side. Most of the windows face the hospital; but it is intended to have a trellis-screen, railing, and gate, by means of which the nurses' garden will be entirely separated from that part of the grounds used by the patients. On the entrance floor there are two large sitting-rooms, one for the sisters and the other for the nurses, and these, in addition to being heated by hot-water coils (this system being carried out all over the building), have tiled fireplaces. There are no fireplaces in the bedrooms, four of which are on this floor, which contains besides a cloak room, and a bright little kitchen with a dresser and gas stove for tea-making, etc. From the passage leading to the nurses' bay-windowed sitting-room a door leads out to an alcove, large enough to be used as a summer sitting-room, and this will be furnished with easy chairs, tables, etc. A verandah on the floor above has been similarly provided; and both are at the back of the home, looking out over the roofs of Chelsea.

Large rugs, writing-tables, and easy chairs, are being supplied for the sitting-rooms, while the bedroom furniture will be of the simplest, including a spring bedstead, combined dressing-table, washstand, and chest of drawers, with a large rug laid on the polished pitch pine floor. Each nurse will have her name on a card, slipped into a neat little brass frame on the door of her bedroom, and will be supplied with a key, the maids, of course, having pass-keys. Buff is the colour chosen for the rooms on one side of the home, and pale blue-green for those on the other. The passages, entrance-hall, etc., are tesselated, and have rounded corners; the walls are cream-coloured, the dado, doors and woodwork being painted a rich mahogany. The four dainty-looking bath-rooms are lined with pale green opalite tiles, and convenient linen and airing cupboards have been supplied. The top floor will be devoted to the Sisters, and there are 25 bedrooms in all. The rooms are ventilated by means of silent mica valve ventilators, and there are fan lights over the doors. The home is lighted throughout by electric light, which can be controlled from a Sister's bedroom, thus avoiding the necessity of going downstairs to ensure lights being out for the night. The home will be occupied from the date of the opening ceremony.

### Appointments.

[No charge is made for announcements under this head, and we are always glad to receive, and publish, appointments. The information, to insure accuracy, should be sent from the nurses themselves, and we cannot undertake to correct officia announcements which may happen to be inaccurate. It is essential that in all cases the school of training should be given.]

Camberwell Infirmary.—Miss E. M. Bradley has been appointed sister. She was trained at the Southwark Infirmary, Dulwich, and has since done private nursing at Anerley.

City Hospital, Park Hill, Liverpool.—Miss J. W. Campbell has been appointed assistant matron, and Miss Annie Dodd and Miss Maria Keywood, charge nurses. Miss Campbell was trained and was for five years at the City of Glasgow Hospital, Belvedere, and she has since been charge nurse and housekeeper at the City Hospital, Park Hill, Liverpool. Miss Dodd was trained at the Royal Infirmary, Sheffield, and has since been assistant nurse at the City Hospital, Park Hill, Liverpool; staff nurse at Kettering General Hospital; staff nurse at Birmingham General Hospital, and private nurse in Derby. Miss Keywood was trained at Fir Vale Infirmary, Sheffield, and the City Isolation Hospital, Nottingham, and has since been ward sister at Monsall Hospital, Manchester; and Queen's nurse at Warsop, Notts.

Dudley Union.—Miss Gertrude Isabel Whiffen has been appointed superintendent nurse. She was trained at Lambeth Union Infirmary. She has been female industrial trainer at Bradfield and Rochford Unions, assistant nurse at Rochford Union, and charge nurse at Great Yarmouth Union Infirmary.

Horton Asylum (London County Council).—Miss A. Nellie Eastman has been appointed matron. She was trained at Southwark Infirmary, Dulwich, was assistant matron at Horton Asylum for a time, and has been matron of the Epileptic Colony, Ewell.

Hospital for Consumption, Brompton.—Miss Alicia Lloyd-Still has been appointed lady superintendent. She was trained at St. Thomas's Hospital, and has since been ward sister and sister at St. Thomas's Home for Paying Patients.

Portsmouth Workhouse Infirmary.—Miss Maude Rodgers has been appointed assistant matron. She was trained at Birmingham Infirmary, and has since been ward sister at Poplar and Stepney Sick Asylum, nurse at the British Hospital, Algiers, and ward and theatre sister at St. Pancras Infirmary (South), Pancras Road, London.

St. George's Infirmary, Fulham Road, London.—Miss Laura Taylor has been appointed home-sister; Miss C. Fitzmaurice night superintendent; and Miss A. E. Cawood sister. Miss Taylor was trained at St. Bartholomew's Hospital, London, and has since been sister at York County Asylum, and temporary matron at Sandgate Sanatorium and Victoria Hospital, Kingston. Miss Fitzmaurice was trained at the City of London Infirmary. She has since been charge-nurse in the same institution, and night superintendent at Hendon Sick Asylum. She holds the L.O.S. certificate. Miss Cawood was trained at the Middlesbrough Infirmary, has since been charge nurse in the same institution, and sister at the Nottingham Infirmary.

Sanatorium, Kingston-upon-Hull.—Miss Sarah Hulton has been appointed sister in charge. She was trained at the Royal Hospital for Sick Children, Edinburgh, and has since been nurse at the Hull Evan Fraser Hospital.

Stockport Infirmary.—Miss Amy Lander has been appointed sister. She was trained at the Royal Infirmary, Preston, and has since been theatre sister in the same institution, and subsequently sister at the Royal Isle of Wight County Hospital, Ryde.

Stone Joint Hospital.—Miss Dean has been appointed nurse-matron. She was trained at Manchester Royal Infirmary, and has since been attached to the North Staffordshire Nursing Institute, and district nurse at Trentham.

The Charterhouse, Hull.—Miss Lena Jordan has been appointed matron. She was trained at Kidderminster Infirmary. She has since been engaged in private nursing.

Union Infirmary, Hope, near Eccles.—Miss Ada Williamson has been appointed charge sister. She was trained at Crumpsall Infirmary, Manchester, and has since been night sister at the Homœopathic Hospital, Birmingham, sister at Sheffield City Hospital, and sister at Crumpsall Infirmary.

### Everybody's Opinion.

[Correspondence on all subjects is invited, but we cannot in any way be responsible for the opinions expressed by our correspondents. No communication can be entertained if the name and address of the correspondent are not given as a guarantee of good faith, but not necessarily for publication. All correspondents should write on one side of the paper only.]

### INSPECTORSHIPS.

Sir William Chance, author of "Children under the Poor Law," writes from Orchards, near Godalming: In an article under this title in your issue of September 24th last a charge of failure of duty is made against an inspector of boarded-out children in that "in one terrible case" she did not thoroughly inspect a child by undressing it, although "the body of the unfortunate mite was subsequently found to be black and blue from frequent thrashings." This might possibly have happened in the case of an inspector of a voluntary association for boarding-out children (*e.g.*, a large mass of children are boarded out by Dr. Barnardo and the Waifs and Strays Society), but I can hardly believe that the incident could have happened under the careful and stringent inspection of the lady inspectors of the Local Government Board. Miss Ward, the Lewis Inspector of Boarding-out, has over and over again pointed out the necessity of examining the bodies of the children to see if they have been subjected to cruel treatment, and the other two lady official inspectors are equally careful. As a strong advocate of efficient and thorough inspection of boarded-out children I think it should be stated, if the fact is so, that you were not referring to an inspector of the Local Government Board, or, if there is any foundation for the statement, the facts of the case should be given so that they may be answered or explained. Knowing the lady inspectors of boarding-out as I do, I cannot believe that any of them could have been guilty of the failure of duty alleged.

[Sir William Chance rightly judges that we did not refer in the "Outlook" on "Inspectorships" to a case connected with the Local Government Board.—Ed. Hospital.]

### EXCESSIVE TRAINING AT A CHILDREN'S HOSPITAL.

"A Graduate of the Children's Hospital Training School," Boston, U.S.A., writes: The writer of the note headed "Excessive Training at a Children's Hospital," in The Hospital of September 17th, appears to be in possession of but part of the facts regarding the training received by nurses in the Children's Hospital Training School of Boston, U.S.A. The period of training has not been extended by the six months occupied in theoretical study and practical instruction, but has been rearranged according to the lines of latest progress in nursing education—that of instructing the pupil in theory previous to actual care of patients. By this method the nurse receives the bulk of actual class instruction in the principles of anatomy, physiology, bacteriology, medical chemistry, hygiene, sanitation and dietetics at a time when she is able to give her undivided effort and attention to these subjects. Moreover, her hospital duties later are not only unhampered by the necessity of attending such lectures and preparing recitations when already physically and mentally exhausted from a day's duty in the wards, but her nursing progress is facilitated by her previous instruction in principles, which enables her to more readily grasp the significance of actual facts. The new method is based upon the modern system of educating from principle to practice, instead of the old laborious and incomplete course of proceeding through vicarious practice to principle. After this six months' course of instruction and study is finished, the pupils spend eighteen months in actual hospital nursing work, surgical and medical, advancing through positions of increasing responsibility in wards and operating-room, with accompanying lectures and clinical instruction upon the subjects concerned. The nurse, having then become grounded in the principles of nursing and actually experienced in all classes of children's diseases, completes her course by spending eight months of her third year in training in adult nursing, part of the time at a large public general hospital and part in a large private adult hospital, in addition to which four months of this year are spent in district nursing, one of the departments connected with the ho[illegible]ital. The criticism that the three years' training only fi[illegible] pupils to nurse children is refuted by the fact that the g[illegible]duates of the Children's Hospital Training School are in qu[illegible] as much demand for adult nursing as are the graduates of training-schools connected with the largest general hospitals.

### CERTIFICATES.

The Matron of the Royal Cornwall Infirmary, Truro, writes: In reference to the article on "Certificates" in your "Outlook" of October 15, I beg to state that a certificate as follows has been given to the nurses of this infirmary since October 1902:—

Royal Cornwall Infirmary, Truro.

This is to certify that —— entered as a probationer in this hospital on the —— day of ——, that she has received three years' training in nursing medical and surgical cases; and that she is fully qualified to efficiently perform the duties of a nurse. Her conduct has been ——. Signed by the Chairman, Matron, and Members of Honorary Medical Staff. [The certificate is on parchment].

### CERTIFICATES AND REGISTRATION.

"Hey What" writes: The list of certificates in the "Nursing Outlook" of October 15th, has suggested to me an argument in favour of registration. The argument had occurred to me before, but the article has encouraged me to produce it. I have for some years been in the position of an employer of nurses and a nurse myself. So I know the ins and outs of both sides of the question. An argument continually used against the need of registration is that the public have only got to get the necessary testimonials from the training schools, and that it is their own fault if they do not protect themselves in this way. But the public do not know what necessary testimonials are, nor what are the proper training schools. I well remember before I had anything to do with hospitals, being greatly puzzled when selecting a district nurse, over the relative merits of provincial hospitals and maternity training places, and for the ordinary lay person, there was no means of finding out whether a certain small hospital was good or not. I fancy that a large proportion of the ladies in local committees do not even know the difference between midwifery and monthly training, and we once very nearly got a monthly nurse as midwife. As to testimonials, the public, as a rule—and naturally—thinks that nothing can be better than a number of glaring testimonials from medical men. Every trained nurse knows that though these are often well merited, they are often very easily obtained. A good-natured house surgeon will not refuse to write one for the nurse with whom he has got on capitally in the receiving-room. I have even known a staff physician ask who the nurse was who had asked him for a testimonial, and for whom he subsequently wrote one. Both these women were "Good sorts," but quite inefficient. Registration seems the only way of protecting the poor, innocent public.

### CLASS LEGISLATION.

"Inquirer" writes: Acting on your suggestion in your "Nursing Outlook" of October 1st, I obtained the Report of the Select Committee of the House of Commons on the Registration of Nurses and the Minutes of Evidence and find, as you say, that nurses trained in infirmaries and small hospitals cannot register if the Bills pass. I have glanced down the list of names of those who belong to the Society of State Registration for Nurses and, to my surprise, I find there some who have been trained at infirmaries and small hospitals and who would not be eligible for registration. If I am wrong perhaps someone will put me right on this point. It may be that the nurses themselves are so keen on the subject that they are willing to be sacrificed. Or is it possible that they are not aware that, should the Bills pass, after their trouble and paying a small subscription to further the cause, they may find that they are no longer required and will not reap the benefit of their labours?

### GOOD NURSING.

"Ward Sister" writes: May I give my way of preparing a patient for operation? I quite agree with "Night Sister" that, if possible, the patient should have a peaceful night before undergoing his operation, and to some extent we can make this easier by giving the necessary purgative about noon the preceding day. I find that by administering a good aperient—Ol. Ricini ℥ii., for instance—about 3 P.M., and a simple enema 12 or 13 hours after, the patient has not at all an uncomfortable night. Regarding nourishment, my patient is not given any food whatever after midnight, unless expressly ordered by the surgeon. I have had my present wards for a year and six months, and have always found this method successful, never having had a case of "collapse or exhaustion," and very little, when any, vomiting. Very often I do not know in time to act on the above lines, having perhaps only six hours' notice of the operation; in which case no food or nourishment of any kind is given, but a simple enema is administered two hours before the surgeon's arrival, and so far everything has been satisfactory. Of course in a child's case a much smaller aperient is necessary, and for a neurotic person it is perhaps wiser to omit the purgative, giving an enema in the evening as well as the early morning one.

### Wants and Workers.

The nurse to whose sad case Nurse Foster, of All Hallows Nursing Home, 127 Union Street, Borough, called attention in our issue of October 8th, begs to thank two readers who have responded to the appeal.

### Echoes from the Outside World.

### Movements of Royalty.

FOLLOWING a luncheon party given on Thursday last by the King to a number of British and American naval and military officers—at which his Majesty proposed the health of the President of the United States and wished prosperity and success to the American Navy—there was an inspection of the Royal Engineers at Chatham on Friday. The houses along the route were decorated, although the occasion being of a semi-private character, there was no public ceremony. The Queen arrived from Copenhagen at Charing Cross on Sunday evening at a quarter past six. The King was present at the station to receive her Majesty, and on his arrival was accorded an enthusiastic reception by a large crowd which had assembled at the barriers. The Queen, who wore a dark travelling costume, and looked in extremely good health, was accompanied by Princess Victoria, Princess Charles of Denmark, and her infant son Prince Alexander. The Duke of Connaught was able to leave Edinburgh for Gosford House on Tuesday.

### Extraordinary Outrage by the Russian Baltic Squadron.

FISHING vessels which arrived at Hull on Sunday evening reported that early on Saturday morning an extraordinary outrage was perpetrated by the Russian Baltic Squadron in the North Sea. It appears that about 1 A.M. on Saturday some 50 of the fishing vessels were at work 220 miles east by north of Spurn Head when the Russian warships came in sight. In the first instance some torpedo-boats approached the fishing fleet, but did not board them. Then the larger ships turned on their searchlights and immediately afterwards opened fire. One steam trawler, the *Crane*, received injuries which left her in a sinking condition; the captain and one of the crew were decapitated by a Russian shot, and several others of the crew were more or less severely injured. Other vessels of the fleet were damaged by round shot or shells, and the incident has created an immense sensation in all parts of the world. On Monday morning the King addressed to the Mayor of Hull a message to the effect that he had heard with profound sorrow of the unwarrantable action which had been committed against the North Sea fishing fleet, and desired to express the deepest sympathy of the Queen and himself with the families who had suffered from this lamentable occurrence. His Majesty has subscribed 200 guineas to the fund for the families of the victims and the Queen £100. In the course of the afternoon Lord Lansdowne was summoned by the King—who desired to see him on the question—and drove to Buckingham Palace, where he had an interview with his Majesty. Subsequently, the Foreign Office issued an announcement that it had been in communication with the representatives of the fishing industry of Hull and Grimsby, and had obtained from them a full statement of the facts. Urgent representations based on this information had, it was added, been addressed to the Russian Government at St. Petersburg, with an intimation that the situation does not admit of delay. On Tuesday the Tsar sent a message through the British Ambassador at St. Petersburg expressing his sincere regret to King Edward and to the British Government for the sad loss of life that had occurred, and intimating that he would take steps to afford complete satisfaction to the sufferers as soon as the circumstances of the case were cleared up.

### The War in the Far East.

ACCORDING to the report of Marshal Oyama, the Russian losses in the Battle of the Sha-ho, as ascertained to October 22nd, are: prisoners, about 700; Russian corpses, 13,333; guns, 45; shells, 6,920; rifles, 5,474; ammunition, 78,000. The corpses have all been buried with military honours. The total Russian casualties during the ten days are estimated at 60,000, and the total Japanese at 30,000. Marshal Oyama reports that 15,879 Japanese officers and men were killed, wounded and missing in the Battle of the Sha-ho. General Kuropatkin states that the Japanese retired on Thursday from Sha-ho village, and General Sakharoff reports that the retreat was precipitate, and that the Japanese left behind a quantity of arms, ammunition, and provisions. By an Imperial Ukase dated Sunday, General Kuropatkin has been appointed Commander-in-Chief.

### Return of the Primate.

THE Archbishop of Canterbury arrived in England on Saturday evening, after his tour of two months in Canada and the United States. He was welcomed at Liverpool by the bishop of the diocese and a number of the clergy, who accompanied him in an informal procession to the river-side railway station. Dr. Davidson declined to be interviewed, but, leaning from his saloon window, he addressed the impromptu congregation, expressing his gratitude for the cordiality of the welcome extended to him. His Grace said that in America he had been received with unbounded hospitality and wonderful kindness. He hoped and believed that his visit to the States might do something to weld together not churches only, but nationalities as well. He came back thankful for all that had passed, having survived all the kind hospitality, and even a railway accident, as part of the record.

### Lord Roberts at Ladysmith.

AFTER climbing to the bastion on the peak of Spion Kop, Lord Roberts was on Friday last week entertained to a banquet by the Siege Club at Ladysmith, the president of the club, in proposing the health of the guest of the evening, referred to the fact that it was the anniversary of the battle of Elandslaagte and the retirement on Dundee. Lord Roberts in his reply stated that the defence being in charge of his old friend, Sir George White, he never doubted that the safety of Ladysmith was assured, and that everything would be done that was possible. He proceeded to say that it gratified him to know that the salvation of Ladysmith during the siege was largely due to the addition of 1,700 volunteers to the Imperial forces; and to express the pleasure which he had experienced in seeing the scene of the siege. It had enabled him to realise how close the enemy had been to the town during the investment, and what that glorious defence meant.

### Death of Lady Dilke.

ON Monday morning Lady Dilke died suddenly at Pyrford Rough, Woking. Born at Ilfracombe in 1840, Emilia Frances Strong married, when she was 22, the late Mark Pattison, rector of Lincoln College, Oxford, who was many years her senior. She shone in Oxford society after her marriage as one of the most remarkable personalities of the time and place. Subsequently, she made a mark as an art critic, and later on as the accomplished and learned historian of French art in the eighteenth century. Pattison died in July 1884, and towards the close of 1885 his widow married Sir Charles Dilke, with whom she had been friends from childhood. She continued to devote herself to the study of art and its history, and found time to take an interest in social and political questions. Her death is deeply regretted by a large circle of friends.

### A Book and its Story.

MR. CHARLES MARRIOTT'S NEW NOVEL.*

Mr. Charles Marriott's new heroine, "Genevra Joslin," is a striking creation, which will add to the author's repute as a writer who has given us more than one clever feminine impersonation. He has also an artist's appreciation of colour and tone in landscape, and a keen perception of beauty is apparent in the care with which every detail is noted, which will serve to brighten the artistic effect of a description. In the present novel the family affairs of the Joslins, Genevra, and her weak-minded brother George, who are the sole survivors of an ancestry which has lived on the Trecoth estate for centuries, are the foundation of the romance. The family of de Jocelin was an old county family in Cornwall dating back to the Norman Conquest, and although the present owner of Trecoth Manor House was but the son of a yeoman, the instinct of race was a dominating one in his sister Genevra, and one of her earliest recollections was connected with a visit to the church, where the de Jocelin tombs were, and her father's injunction that she was ever to bear in mind the stock from which she had sprung. At the age of twenty-nine, we read: "Genevra had found her only emotional escape in writing verse. This was practised furtively, for although her poetry was so definite and apart, so functional, that it was never menaced by the practical duties of life, she was yet more business-like, and less given to day dreaming, than her brother. She cultivated her talent so unobtrusively, that beyond remarking that her name occasionally appeared in print, her immediate associates mercifully ignored her as a poet. Socially, her position was peculiar and under the circumstances fortunate. She was the daughter of a yeoman farmer and lived with her brother at the old Manor House of Trecoth. Consequently she was kept out of drawing-rooms and in daily contact with the more essential facts of life, but she allowed her family pride to govern all her intercourse with other people." As sedulously as Genevra cultivated her "incurable habit" of poetry she cherished in secret, also, the ruling ambition of her life, the restoration of the present half ruinous remains of the once beautiful mansion of Trecoth to something like its original dignity. This was to be effected, either by the accumulation of money by her own personal effort, or by marriage. She had, through her contributions to a monthly review, "distinguished by reason of its austerity" come into contact with the editor Edgar Noy, himself a poet, "notorious throughout the literary world for his indifference to accepted opinions and the pungency of his criticisms on contemporary writers." He was one of the few enthusiasts who recognised Genevra's peculiar gift, and prophesied a future for her. "Brains? It isn't brains, it is woman," he had said once, in discussing her work with the only man of letters who remained his friend, adding, that "marriage would be fatal to her career as a poet unless—her husband deserted her in a week." Genevra's position at Trecoth was not an easy one at this time. She had lived happily enough as her brother's housekeeper and general assistant for two years after their father's death, and her frugal housekeeping and actual help in the fields at "tealing time" together with the quiet encouragement which she gave to George enabled him to keep affairs on the farm in a fairly prosperous condition. Perseverance was not, as with his sister, a marked characteristic, and he made the first step toward the road which ultimately ended in disaster by marrying his cousin; a woman whose narrow mind and jealous temperament made Genevra's life almost unendurable.

By the terms of their father's will George came into possession of the Trecoth Estate on one condition only, that he gave a home and one-third of the income arising from the place to Genevra, until she married. This is the condition of things when the book opens. George, to add to his complications, had, upon the persuasion of his wife, bought a flower-farm with money borrowed from an old schoolfellow, Sampson Oliver, giving as security the title-deeds of the estate, hoping by the profits from the flower venture to be able to redeem them, before the time of mortgage—ten years—expired. "Had George been a man of industry and enterprise it is probable that in time he would have made a comfortable income from the early narcissi and daffodils, but a succession of cold springs by which the flowers were delayed and the market price lowered . . . hampered him from the outset. He was easily discouraged, he lacked the knowledge to make the most of his resources, and his wife, jealous of Genevra, robbed him of the intelligent help she might have given him."

Genevra, as she stands for the reader's inspection, is thus described at this juncture. "It would have been as difficult for an observer to place Genevra Joslin socially, as to tell her age and condition. Her walk, her mature figure, and the gravity of her expression suggested the married woman, until one saw the virginal freshness of her face, and comprehended that the seriousness was from within, and not the result of a disappointing experience of life. Obviously she dressed with care, choosing her clothes with a keen and just appreciation of her physical characteristics. . . . Thus the details of her dress were so considered that they expressed a personality. Her eyes were brown, large, and steadfast, with subtle drawing in their lids; her eyebrows well defined, neither straight nor arched, but with a delicate lift at their outer extremities. Her whole person suggested ripeness, readiness, as of wine slowly matured; in another year or two she would be past her prime. . . . A hasty observer would perhaps have set down Genevra as probably a very selfish woman, if not absolutely heartless. A nearer glance into her eyes, full of sleeping passion, haunted with ideas, must have awakened the interested belief that she was still waiting for a crisis; that by some fault of circumstances, not from insensibility, a singularly full inner life had as yet failed to find an emotional object upon which to spend its emotional richness." The "crisis" for which Genevra was waiting comes before very long and is brought about, by the irony of circumstances, by her sister-in-law's introduction into the household of Trecoth, of an artist, Leonard Morris, to whom she lets two rooms. By this time George Joslin, unable to pay his way, has borrowed considerably of Genevra, and she, to save the honour of their name, and to keep the manor in the family, has reluctantly promised to marry Oliver Sampson. Her reception of his attentions makes very good reading. But every other interest is subservient to the one which follows the arrival of the strong man at Trecoth. Through repulsion to attraction would best express the drawing together of the artist and poet. But in spite of the leisured development of their love, it was not without its effect on Genevra at least, for we read that when her book of poems appeared, the editor and his friend, commenting upon their beauty, again discuss Genevra: "This is altogether different," "I wonder who helped her?" said Noy, "I wonder who helped her to mature?"

* "Genevra." By Charles Marriott. (Methuens. 6s.)

### Notes and Queries.

**FOR REGULATIONS SEE PAGE 12.**

*Home.*

(28) Will you kindly tell me of a home where an epileptic lady could be received at not more than 10s. a week ?—*Nurse.*

The Meath Home of Comfort, Westbrook, Godalming, or St. Luke's Home for Epileptic Churchwomen might suit. Write as to the latter to the Deaconess, 36 Parkwood Road, Bournemouth.

*Canada.*

(29) Will you kindly let me know where I can obtain information regarding nursing in Canada, both private and hospital ?—*Anxious.*

I am very anxious to get a post as private or district nurse in Canada. Can you tell me to whom to apply for particulars ?—*Nurse J. C. M.*

The Lady Superintendent, the Victorian Order for Nurses, 578 Somerset Street, Ottawa, Ontario, Canada, might be able to advise.

*Maternity Taining.*

(30) I should feel obliged if you will give me the name of an institution in London where I could take a course of training for the L.O.S. examination. I am a trained nurse, but not in maternity.—*I. P.*

For list of the lying-in hospitals see "The Nursing Profession: How and Where to Train."

*Registered.*

(31) I was trained for one year in hospital and since then I have been nursing for a private institution; will you kindly tell me if I need to be registered, and, if so, to whom should I apply ?—*Nurse M.*

There is no State registration for general nurses.

*Factory and Sanitary Inspectors.*

(32) Can you inform me the necessary qualifications for the position of factory inspector? Also, if one can qualify by a postal course for the examination of the Sanitary Institute? I should also be glad to get any information in regard to the prospects of nurses in this branch of the work.—*F. R. S.*

Details of the qualifications for the position of factory inspector may be obtained on application to the Under-Secretary, Home Office, S.W., but there are seldom vacancies. For full particulars as to the conditions on which the certificate of the Sanitary Institute is given write to the Secretary, Sanitary Institute, Margaret Street, W. We are afraid that the supply for women inspectorships exceeds the demand.

*Cottage Hospital Certificate.*

(33) Would a certificate from cottage hospital of twelve beds qualify candidate as nurse in good general hospital or nursing institution.—*Hon. Sec.*

No, it would only prove that she had received preliminary training.

*Sea-Going Nurses.*

(34) Can you tell me where I should have to apply to obtain a post of nurse on board a ship. America line preferred if they take nurses.—*F. O'C.*

You might write to Miss Kate Penn, Twentieth Century Club, Stanley Gardens, W., but we are afraid that at present such a post is not obtainable.

*Queen's Nurses.*

(35) Will you kindly give me information concerning "Queen's Nurses," what qualifications are necessary, and what advantages they enjoy ?—*M. S.*

Write for the regulations to the Secretary, Queen Victoria's Jubilee Institute for Nurses, 120 Victoria Street, S.W.

**Handbooks for Nurses.**

"The Nurses' Pronouncing Dictionary." 2s. post free.

"Surgical Ward Work and Nursing." By Dr. A. Miles. Price 3s. 6d. net; 3s. 10d. post free.

"The Human Body: Practical Physiology and Hygiene." 5s. post free.

"The Nursing Profession: How and Where to Train." 2s. net; 2s. 4d. post free.

"Preparation for Operation in Private Houses." Price 6d. post free.

The above may be obtained of all booksellers or of The Scientific Press, Limited, 28 & 29 Southampton Street, Strand, London, W.C.

### For Reading to the Sick.

ALL SAINTS' DAY.

Of all the thoughts of God that are
Borne inward into souls afar,
Along the Psalmist's music deep,
Now tell me if that any is
For gift or grace surpassing this—
"He giveth His belovëd sleep"?

. . . . . . .

For me my heart that erst did go
Most like a tired child at a show,
That sees through tears the mummers leap,
Would now its wearied vision close,
Would childlike on His love repose
Who giveth His belovëd sleep.

. . . . . . .

And friends, dear friends, when it shall be
That this low breath is gone from me,
And round my bier ye come to weep,
Let One, most loving of you all,
Say, "Not a tear must o'er her fall!
He giveth His belovëd sleep."

*E. B. Browning.*

One great miracle in the new creation of God is this, that death is changed to sleep; and therefore in the writings of the New Testament we do not read of the "death" of the saints.

St. Paul speaks of the saints unseen as of those that "sleep in Jesus"; and Christians were wont to call their burial-grounds cemeteries, or sleeping-places, where they laid up their beloved ones to sleep on and take their rest. Let us see why we should thus speak of those whom we call dead.

First it is because we know that they shall wake up again. What sleep is to waking, death is to the resurrection. It is only a prelude, a transitory state, ushering in a mightier power of life; therefore death is called sleep, to show that it has a fixed end coming. . . . Let us be much in thought with them that are at rest. They await our coming; for without us they shall "not be made perfect."—Heb. xi. 40. Let us, therefore, remember and love and follow them; that when our last change is over we, with them, may "sleep in Jesus."—*H. E. Manning.*

To the soul standing with all its questionings before the door of the tomb, He who liveth and was dead comes as He came to Martha saying "Thy brother shall rise again." If we believe in Him, we do believe those words, and death is really changed to us, and the dead are really living by the assurance of the living Christ. It is a beautiful connection, one whose mysterious beauty we are always learning more and more, that the deeper our spiritual experience of Christ becomes, the more our soul's life really hangs on His life as its Saviour and continual Friend, the more real becomes to us the unquenched life of those who have gone from us to be with Him. In those moments when Christ is most real to me, when He lives in the centre of my desires and I am resting heavily upon His help, in those moments I am surest that the dead are not lost, that those whom He in whom I trust has taken He is keeping. The more He lives to me the more they live.—*Phillips Brooks.*

O God of Mercy, grant that when we lie down in the last long calm sleep of death we may commit ourselves trustfully into Thy hands, and that on the morning of the Resurrection we, with all Thy faithful departed, may be perfected in that day when Thou makest up Thy jewels. Of Thy Mercy, O our God, Who art blessed and livest and reignest for ever and ever. Amen.—*E. Vaux.*

